# Effectiveness of app-based relaxation for patients with chronic low back pain (Relaxback) and chronic neck pain (Relaxneck): study protocol for two randomized pragmatic trials

**DOI:** 10.1186/1745-6215-15-490

**Published:** 2014-12-15

**Authors:** Susanne Blödt, Daniel Pach, Stephanie Roll, Claudia M Witt

**Affiliations:** Institute for Social Medicine, Epidemiology and Health Economics, Charité - Universitätsmedizin - Berlin, Berlin, Germany; Institute for Complementary and Integrative Medicine, University Hospital Zurich and University Zurich, Zurich, Switzerland

**Keywords:** Neck pain, Low back pain, Relaxation, Mind-body therapies, Smartphone application, Comparative effectiveness research

## Abstract

**Background:**

Chronic low back pain (LBP) and neck pain (NP) are highly prevalent conditions resulting in high economic costs. Treatment guidelines recommend relaxation techniques, such as progressive muscle relaxation, as adjuvant therapies. Self-care interventions could have the potential to reduce costs in the health care system, but their effectiveness, especially in a usual care setting, is unclear. The aim of these two pragmatic randomized studies is to evaluate whether an additional app-delivered relaxation is more effective in the reduction of chronic LBP or NP than usual care alone.

**Methods/design:**

Each pragmatic randomized two-armed study aims to include a total of 220 patients aged 18 to 65 years with chronic (>12 weeks) LBP or NP and an average pain intensity of ≥ 4 on a numeric rating scale (NRS) in the 7 days before recruitment. The participants will be randomized into an intervention and a usual care group. The intervention group will be instructed to practice one of these 3 relaxation techniques on at least 5 days/week for 15 minutes/day over a period of 6 months starting on the day of randomization: autogenic training, mindfulness meditation, or guided imagery. Instructions and exercises will be provided using a smartphone app, baseline information will be collected using paper and pencil. Follow-up information (daily, weekly, and after 3 and 6 months) will be collected using electronic diaries and questionnaires included in the app.

The primary outcome measure will be the mean LBP or NP intensity during the first 3 months of intervention based on daily pain intensity measurements on a NRS (0 = no pain, 10 = worst possible pain). The secondary outcome parameters will include the mean pain intensity during the first 6 months after randomization based on daily measurements, the mean pain intensity measured weekly as the average pain intensity of the previous 7 days over 3 and 6 months, pain acceptance, ‘LBP- and NP-related’ stress, sick leave days, pain medication intake, adherence, suspected adverse reaction, and serious adverse events.

**Discussion:**

The designed studies reflect a usual self-care setting and will provide evidence on a pragmatic self-care intervention that is easy to combine with care provided by medical professionals.

**Trial registration:**

ClinicalTrials.gov identifier Relaxback NCT02019498, Relaxneck NCT02019134 registered on 18 December 2013.

## Background

Low back pain (LBP) and neck pain (NP) are highly prevalent conditions in industrialized countries with an estimated lifetime prevalence of 84% [1] and 30 to 70%, respectively [2, 3]. Of 281 conditions investigated in the Global Disease Study 2010, LBP ranked first and NP ranked fourth on a measure of years lived with disability in Western Europe [4, 5]. The global point prevalence of LBP was 9.4% (95% CI 9.0 to 9.8) [6] and that of NP was 4.9% (95% CI 4.6 to 5.3) [7]. In a representative survey of the German population from 2009, 20.7% of the respondents reported to have had LBP for at least 3 months in the previous year [8]. Work absence, disability and medical costs related to LBP and NP generate a high individual and societal burden [4, 9, 10]. Individuals suffer from a decrease in their quality of life and societal ramifications include absenteeism and early retirement [10, 11]. In Germany, the annual costs (direct and indirect) of back pain have been estimated at 16.4 to 50 billion Euro (approximately 2.2% of the gross domestic product in 2006) [12]. Although the majority of patients experience pain relief within 3 months, approximately 5 to 20% of individuals with LBP [13] and NP [14, 15] will develop sub-acute and chronic LBP. Numerous LBP and NP treatments exist, although available evidence suggests no more than moderate effects from most of the available treatments [16, 17]. Pain experts agree that most pain is best treated using a combination of medication with a self-management, complementary, non-drug therapy [18]. Therefore, in the last decade, multimodal treatment strategies have been established by national and international guidelines combining education, physiotherapy, medication, self-care and behavioral therapies [17, 19–23]. In the German national guidelines on LBP, paracetamol, NSAIDs and opioid analgesics (not longer than 3 months) are recommended as drug therapy. Physical activity, relaxation, ergotherapy, manipulation/mobilization, education, and behavioral therapies are recommended as non-drug therapies for chronic LBP [19]. Chronic NP should be treated with physiotherapy, and if necessary, in combination with manual therapy, post-isometric relaxation and muscle strengthening, and intramuscular injection of lidocaine. Other therapies based on patient preferences could be considered but cannot be recommended based on strong evidence [17]. The integration of active self-care therapies, such as ‘mind-body therapies’, for chronic pain treatment is supported by evidence [24]. Additionally, self-care interventions are highly attractive because they could reduce expenses for health care systems, are relatively safe, and might have health benefits beyond the effects on pain [24–27]. ‘Mind-body therapies’ including relaxation, meditation and guided imagery are easily applicable as self-care interventions and are used as adjuvant therapies for the treatment of chronic pain [28, 29]. One aim of several mind-body therapies, including relaxation techniques and meditation, is to reduce chronic stress and enhance well-being through the elicitation of the ‘relaxation response (RR)’ the opposite of the body’s stress response to perceived threats [30]. The RR is induced when a person focuses on a word, sound, phrase, repetitive prayer, or movement, and disregards everyday thought [31]. The relaxation response is defined as a physical state characterized by decreased arousal of the sympathetic nervous system [32] and is the opposite of the stress or ‘fight or flight’ response [30, 32]. Progressive muscle relaxation (PMR) has been shown to be an effective treatment for reducing chronic non-specific NP [33] and in Germany, PMR is recommended by the national disease management guidelines for LBP [19] and by the ‘guideline NP’ of the German Society of General Practice and Family Medicine [17]. ‘Mindfulness-based’ therapies have been shown to be effective based on clinical studies on the treatment of chronic pain [34], and a Cochrane review on patients with chronic LBP suggested that therapy using PMR or electromyographic (EMG) biofeedback is more effective than control treatments for pain relief and improving short-term functional status; however, this evidence was of low quality [35]. There has been encouraging evidence from a systematic review that showed that guided imagery can reduce musculoskeletal pain [36] and limited evidence that ‘mindfulness-based’ stress reduction improves pain acceptance [37]. Autogenic training (AT) is a commonly used self-help relaxation technique that is easily applicable in daily life [38]. Most of the mind-body therapies train patients in a group setting, but alternative delivery methods such as audio guidance from a smartphone may be more pragmatic in a usual care setting. Smartphones are increasingly common in the population. In Germany, it was estimated in February 2014 that 40 million people owned a smartphone [39], which is approximately 49.5% of the German population [40]. Coinciding with the popularity and wide utilization of smartphones, the number of available smartphone applications (apps) has increased rapidly [41], and apps are currently used to support people with various health problems. In a search conducted in July 2012, in the official Android, BlackBerry and iPhone Smartphone app platform stores in the United States, 220 pain apps were identified. Chronic non-specific pain was the focus of more than half (55.5%) of the apps followed by back pain and/or NP (25.9%) with the purpose of the apps being pain education (24.1%), pain self-management (62.3%) and both education and pain management (13.6%). Health care professionals were only involved in the development of a small number of these apps (35%) [42]. In a further search conducted between June and August 2010 in the official stores of the five major operating systems (iOS, Android, Blackberry OS, Nokia/Symbian and Windows Mobile) for available apps against pain, headache and migraine were the most commonly targeted conditions, followed by back pain. The reported purpose of the apps was mostly pain relief or educational information about pain. Apps that target pain often include an audio relaxation component. Regrettably, there is limited available information on the effectiveness of those apps as self-care interventions [41]. Furthermore, to date, the effectiveness of additional relaxation on chronic LBP or NP in a usual care setting is unclear. The aim of these two pragmatic randomized studies is to evaluate whether additional app-based delivered relaxation is more effective in the reduction of chronic LBP or NP than usual care alone.

## Methods/Design

### Design

Each study is a two-armed, randomized, parallel group, single center pragmatic trial investigating the effectiveness of additional relaxation exercise for patients with chronic LBP (Relaxback) and NP (Relaxneck) compared to usual care alone. The intervention duration is 6 months, with the primary outcome summarizing the effect in the first 3 months.

An app has been developed for collecting outcome data and to provide the relaxation intervention in the form of audio content to the participants of the intervention group.

As an essential part of comparative effectiveness research [43], stakeholders were included in the planning of the study. In July 2013, one stakeholder meeting included patients with NP (n = 3) and LBP (n = 4), psychologists (n = 2) and clinical researchers (n = 2). Stakeholder involvement resulted in the following resolutions:

- Duration of the intervention was shortened from 20 to 15 minutes daily
- Participants can individually determine the time at which they will receive their push notifications
- Diary content was determined (questions about the intensity of the pain, intensity of ‘LBP-/NP-related’ stress, adherence, and amount of pain medication intake)
- The German translation (Imaginationstraining) of guided imagery was determined.

### Participants

Potential participants will be informed with brochures and posters at universities, colleges, gyms, and doctors’ offices. The studies will include patients aged 18 to 65 years with chronic LBP or NP (≥12 weeks) and a mean pain score of ≥ 4 on a numeric rating scale (NRS) in the previous week. In order to be included in the studies, the patients have to be willing to be randomized and provide written and oral informed consent; furthermore, patients are required to follow study interventions according to the protocol, to complete the baseline questionnaire in paper form as well as electronic questionnaires and diaries provided by a smartphone application. Additionally, having a smartphone (iPhone or Android) is required. Participants must identify LBP or NP as their primary complaint to participate in the Relaxback or Relaxneck study, respectively.

Participants are excluded in case of their LBP/NP being caused by a known malignant disease, trauma, the presence of a known rheumatic disorder, a history or planned surgery of the spinal column of the low back/neck in the next 6 months, known neurological symptoms: for example, radicular symptoms because of a prolapsed disc, regular intake of analgesics (> once per week) because of additional disease, intake of centrally-acting analgesics, or a history of severe acute or chronic disorders that do not allow participation in the therapy. Further exclusion criteria include known alcohol or substance abuse, insufficient German language skills, current application for a pension claim, participation in another clinical trial during the 6 months before the study and parallel to the study or applying regular relaxation techniques, mindfulness meditation or any other ‘mindfulness-based’ therapy 6 weeks before the study or planned in the next 6 months.

### Randomization

For both studies, if a participant meets all inclusion and none of the exclusion criteria, they will be randomized to either the control (usual care only) or the intervention (usual care plus relaxation) group using blocked randomization with variable block lengths and an allocation ratio of 1:1, that is 110:110 participants for each study. The randomization sequence will be generated by a data manager, who will not be involved in the analysis of the data or the enrollment of the patients; SAS (version 9.3, SAS Inc., Cary, NC, USA) will be used for this process. The randomization list will be included in a safe Microsoft Access (Redmond, WA, USA) database to ensure that it is not accessible during the randomization process of individual participants and that screened patients are strictly consecutively enrolled. The randomization process will be conducted by the study office at the Institute for Social Medicine, Epidemiology, and Health Economics. To ensure allocation concealment upon inclusion into the study, the staff will enter participants into the database and will receive the intervention or control group allocations.

### The app

The app includes relaxation audio files, notification features, diaries, and questionnaire options. The app was developed for smartphones running the iOS or Android operating systems. The app concept was approved by the data protection officer of the Charité - Universitätsmedizin Berlin.

### Relaxation intervention

To induce a relaxation response, 3 exercises (AT, mindfulness meditation and guided imagery) with a length of 15 minutes each will be made available in 2 versions (female and male voice). They will be accompanied by a short instructional text. Relaxation exercises can be applied in different positions (sitting, walking and lying), which can be chosen by the participants according to their needs. The relaxation exercise should be applied daily or at least 5 days per week for 6 months.

AT was developed by the German psychiatrist Johannes Schultz in 1932. The AT audio has a focus on the physical sensation of the breath or heartbeat and visualizes the body as warm, heavy and/or relaxed [44]. The AT teaches participants to react to six verbal commands such as ‘my arms are very heavy’, ‘my heart beats regularly and calm’ and ‘my belly is warm’ to make the body feel relaxed.

Mindfulness is a practice rooted in *Vipassana* (translation: insight) meditation, which has Buddhist roots. It is defined as ‘paying attention in a particular way: on purpose, in the present moment and in a nonjudgmental way’ [45]. The mindfulness meditation focuses on the breath and uses the breath as an anchor when the mind starts to wander. This concept is also used in Mindfulness-Based Stress Reduction developed by Kabat-Zinn [45–47].

In guided imagery, the mind is directed to intentionally create images to produce positive changes [48]. The audio guides participants to visualize/conjure a place that is associated with positive feelings such as safety, security and well-being. The guided imagery audio is accompanied by soft background music and directs the visualization and imagination to a pleasant and peaceful place that has meaning for the participant to replace negative or stressful feelings [49].

### Control group

Patients in the control group continue using usual care defined as all medical and non-medical treatments; however, relaxation techniques, mindfulness meditation or any other mindfulness-based training are not permitted.

### Outcome measurement

The primary outcome measure will be the mean LBP or NP intensity during the first 3 months of intervention based on daily pain intensity measurements on a NRS (0 = no pain, 10 = worst possible pain) [50].

The secondary outcome parameters include the following: the mean pain intensity during the first 6 months after randomization based on daily measurements; the mean pain intensity (NRS) measured weekly as the average pain intensity of the previous 7 days over 3 and 6 months; pain acceptance (German version of Chronic Pain Acceptance Questionnaire [51]); ‘LBP-/NP-related stress’; sick leave days; and pain medication intake. Additionally, we will collect data about expectations, adherence, self-reported general changes in LBP/NP, suspected adverse reactions and serious adverse events. The outcome measures including the time points are listed in Table 1.

**Table 1:** **Data collection: except for the baseline data, outcomes are assessed throughout the 6-month period**

	Baseline	Daily ^d^	Weekly ^d^	After third month ^d^	After sixth month ^d^
Socio-demographics (age, migration background, education)	x				
Pain intensity (NRS)		x			
Mean pain intensity in the last 7 days (NRS)	x		x		
Mean perceived ‘LBP-/NP-related’ stress intensity in the last 7 days (NRS)	x		x		
Pain acceptance	x			x	x
Sick leave days	x			x	x
Intake of medication against LBP/NP	x		x		
Suspected adverse reaction^a^				x	x
Serious adverse events				x	x
Application of other therapies	x			x	x
Expectation	x				
Adherence^a^		x^b^	x^c^		
Self-perceived improvement				x^a^	x^a^

^a^Only in the intervention group.

^b^Assessed by tracking.

^c^Assessed by the diary (for relaxation without audio use).

^d^Provided by the app.

Abbreviations: LBP, low back pain; NP, neck pain; NRS, numeric rating scale.

### Data collection

Baseline information will be collected using paper and pencil, and all follow-up data will be collected using the study app.

The study app features electronic daily and weekly diaries in which study participants will log daily data regarding their LBP/NP intensity and weekly data on medication intake, average pain intensity over the previous 7 days based on a NRS (‘How strong was your mean pain intensity in the last 7 days?’), and ‘LBP-/NP-related stress’. Daily and weekly data will be collected throughout the 6-month period. Furthermore, the apps include questionnaires that appear after the end of the third and sixth months.

### Assessment of adherence

Adherence will be assessed by 1) tracking of the running time of the relaxation audios (start and end times, and the type of relaxation) and 2) by asking the participants how much time they spent doing the relaxation exercises without using the app.

### Assessment of safety

Adverse events and suspected adverse reactions (only in the intervention group) are assessed after the third and sixth months using the questionnaire provided by the app.

### Sample size

In the literature, an effect size of 0.62 has been described for mind-body therapies compared to no intervention in a group setting [29]. We assume a smaller effect size of 0.4 (Cohen’s d, baseline adjusted) for individual self-care relaxation exercise compared to usual care alone because individuals might be less focused and consequently less adherent in a self-care setting. To obtain a power of 80% using a 2-sided *t*-test with significance level of 0.05, 100 participants for each treatment group are needed (a total of 200 participants). We will include 110 participants per group (220 in total for each study) to compensate for dropouts.

### Data analyses

For each study, the primary analysis of the primary outcome (mean pain intensity over 3 months measured as the daily pain intensity on a NRS) will be performed using an analysis of covariance (ANCOVA) with a fixed factor of ‘treatment group’, adjusted for the baseline NRS value (fixed covariate). This will be used to test the null hypothesis of equal mean pain intensity between the two treatment groups. The analysis will be based on the full analysis set (FAS, all available data without imputation of missing values, based on the intention-to-treat principle) with a 2-sided significance level of 0.05. The results will be reported as adjusted group means with 95% confidence intervals and the *P-*value for the group comparison. All further analyses on the primary and all secondary outcomes will be considered explorative, and no adjustment for multiple testing will be performed. The secondary outcomes will be analyzed for the FAS similarly to the primary analysis depending on the scale and distribution of the data, that is ANCOVA, logistic regression, or Poisson regression, adjusted for the respective baseline value (when available). For the sensitivity analysis, the primary analysis of the primary outcome will be repeated based on the per-protocol population. In addition, missing data on the primary outcome will be imputed using multiple imputation techniques or other methods depending on the assumed missing data mechanism(s). In case of relevant differences in baseline variables between the treatment groups, the analysis of the primary outcome will be repeated with additional adjustment for these variables. As further supportive analysis, a mixed model for repeated measures (MMRM) will be fitted to compare the two treatment groups with respect to changes in the primary outcome over time. The model will include terms for treatment and time as fixed main effects, an interaction term for treatment by time, and the subject as a random effect, adjusted for the baseline value. The following subgroups will be considered for exploratory analyses: age groups, education (>10 years of school education, ≤10 years of school education), sex (male/female), severity of disease and duration of disease. Subgroup analyses will be performed on the primary outcome by including an interaction term (subgroup variable by treatment) in the main model, as well as by performing separate analyses for each subgroup. A detailed statistical analysis plan (SAP) will be developed prior to any data analyses. Data analysis will be performed in SAS version 9.3 or higher (SAS Inc., Cary, NC, USA).

### Ethics

The protocols of the studies were approved by the local ethics review boards at the Charité - Universitätsmedizin Berlin (approval number: Relaxback EA 1/260/13 and Relaxneck EA 1/259/13), and the studies will be conducted according to common standard guidelines for clinical trials (Declaration of Helsinki, and where it applies, the International Conference on Harmonization of Technical Requirements for Registration of Pharmaceuticals for Human Use and Good Clinical Practice (ICH-GCP) revised version, Somerset West, Republic of South Africa, 1996). All study participants will provide oral and written informed consent.

### Pragmatic explanatory continuum summary

From a methodological point of view, a study can be more focused on effectiveness or efficacy. ‘Effectiveness’ is defined as a measure of the extent to which an intervention, when deployed in the field in routine circumstances, does what it is intended to do for a specific population, whereas ‘efficacy’ refers to the extent to which a specific intervention is beneficial under ideal conditions [52]. The assessment of effectiveness in pragmatic trials can often be more relevant to policy evaluation and the health care decisions of providers and patients than the assessment of efficacy in explanatory trials [53]. Therefore, greater detail and transparency in reporting of the intervention characteristics, settings and assessment specifics are needed [54, 55]. The pragmatic explanatory continuum summary (PRECIS) [56] was developed to provide a graphical tool of ten design domains, which helps researchers categorize a trial’s focus as more pragmatic (effectiveness) or explanatory (efficacy). However, clinical studies are very often placed somewhere in the middle of the pragmatic-explanatory continuum [53, 57].

**Figure 1:**
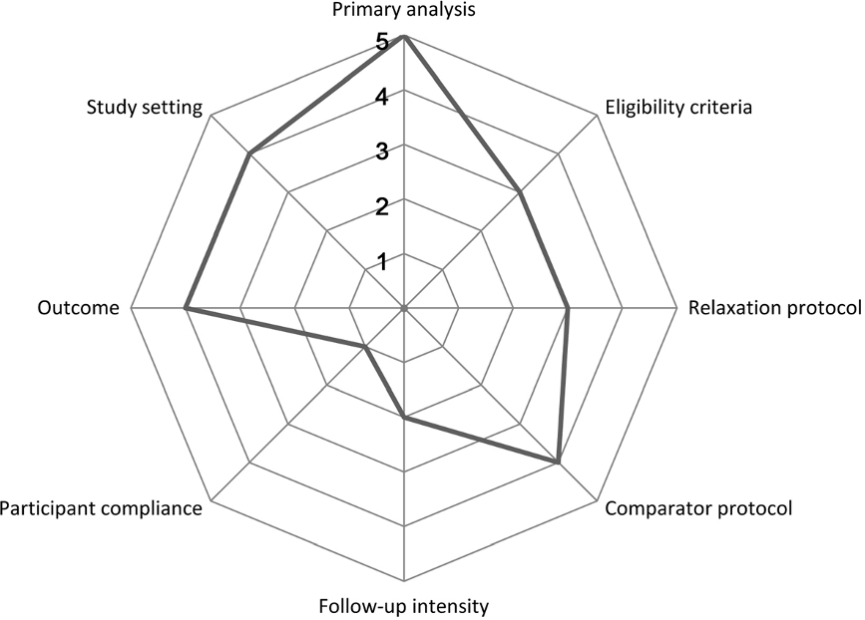
**Pragmatic-explanatory continuum indicator summary (PRECIS) for the main study displaying the location of the eight study design criteria to be more on the pragmatic side (outer circles), more of the explanatory side (inner circles), or in the middle of the continuum.**

Three of the authors (CMW, DP, SB) determined the placement of the study using PRECIS. The place of our trial in the pragmatic-explanatory continuum was defined more on the pragmatic side for the criteria of primary analysis, relaxation protocol, control protocol, eligibility criteria and outcome measurements, whereas participant compliance, follow-up intensity, and relaxation protocol were more on the explanatory side (Figure 1). Based on the ten PRECIS criteria, practitioner expertise and adherence were not applicable, so that 8 study design criteria remained.

## Discussion

To the best of our knowledge, these are the first randomized pragmatic trials that will investigate whether app-delivered relaxations are an effective method to reduce chronic LBP or NP.

Chronic LBP and NP are common problems that constitute a large socioeconomic burden [4, 7, 58], and self-care therapies delivered by an App are easy to apply in a ‘real world setting’. Despite an increasing number of available pain related apps, very few of them have been tested for effectiveness [41]. Therefore, we planned both studies as pragmatic trials.

Due to the similar disease pattern of the two conditions, the intervention and study design is suitable for both patient groups, thus fewer resources than usual were necessary to conduct two parallel trials.

The involvement of relevant stakeholders contributed positively to the development process of the two study protocols. One important result of the stakeholder involvement was the reduction of the exercise duration from 20 to 15 minutes daily because the stakeholders suggested that long exercise time might be associated with difficulty following the recommended protocol: 15 minutes was considered the minimal daily exercise time that is necessary to achieve a treatment effect, especially outside of a group setting, and this length was chosen to improve patient adherence to the exercises. Adherence is a strong predictor of treatment success in patients with chronic LBP [59]. To support adherence, we implemented notifications in the app that can be individualized and remind the participants to perform the exercises. The selection of three different exercises to induce the relaxation response considers the individual’s expectations, beliefs and preferences [60] and contributes to a more personalized, patient-centered therapy.

In these studies, the app will also be used as a tool for data collection. The content and the length of the electronic questionnaires and the diaries were chosen based on our previous experience with an app as a data collection tool in a study on menstrual pain [57]. Broad stakeholder involvement revealed that outcome measurements should be patient-relevant. It also indicated that app-delivered questionnaires can be provided more frequently than paper-pencil questionnaires as the completion of the questionnaires on smartphones requires less time [57]. Consequently, the app includes one daily question, short weekly diaries and two longer questionnaires only after the third and the sixth months. However, although we tried to minimize the number of outcome measurements, the valid tool for assessing pain acceptance [51] (after the third and sixth month) includes 20 questions and had to be implemented. Moreover, questionnaires adapted for app delivery are not yet validated and no data exists on whether the length or the frequency of electronic questionnaires influences the drop-out rates in randomized controlled trials.

We decided to measure pain intensity (NRS) daily and the average pain intensity over the previous 7 days was used to investigate whether there are differences between these 2 methods of pain assessment. This approach takes into account the consistent findings in pain assessment research that have shown that recall ratings of average pain might overestimate pain compared to ‘actual’ pain scores received from daily diaries [61–64], and it might minimize recall bias. Hence, the results of the study will add evidence to the ongoing discussion of the potential utility of recall ratings in pain clinical trials.

The main limitation of the study is that the patients will not be blinded, which might increase bias. It has been reported that bias due to lack of patient blinding is pronounced in complementary/alternative randomized clinical trials with patient-reported outcomes. However, the designed studies reflect a usual self-care setting and will provide evidence for a pragmatic self-care intervention that is easy to combine with care provided by medical professionals.

### Trial status

Recruitment was started in March 2014.

## Abbreviations

Ancova: analysis of covariance
AT: autogenic training
App: smartphone application
EMG: electromyographic
FAS: full analysis set
LBP: low back pain
MMRM: mixed model for repeated measures
NP: neck pain
NRS: numeric rating scale
NSAIDs: nonsteroidal anti-inflammatory drugs
PMR: progressive muscle relaxation
PRECIS: pragmatic explanatory continuum summary
SAP: statistical analysis plan
RR: relaxation response.

## References

[1] Airaksinen O, Hildebrandt J, Mannion AF, Ursin H, Brox JI, Klaber-Moffett J, Reis S, Zanoli G, Cedraschi C, Kovacs F, Staal JB (2004). European Guidelines for the Management of Chronic Non-Specific Low Back Pain.

[2] Bergman S, Herrstrom P, Hogstrom K, Petersson IF, Svensson B, Jacobsson LT (2001). Chronic musculoskeletal pain, prevalence rates, and sociodemographic associations in a Swedish population study. J Rheumatol.

[3] Borghouts JA, Koes BW, Vondeling H, Bouter LM (1999). Cost-of-illness of neck pain in the Netherlands in 1996. Pain.

[4] Hong J, Reed C, Novick D, Happich M (2013). Costs associated with treatment of chronic low back pain: an analysis of the UK General Practice Research Database. Spine (Phila Pa 1976).

[5] Vos T, Flaxman AD, Naghavi M, Lozano R, Michaud C, Ezzati M, Shibuya K, Salomon JA, Abdalla S, Aboyans V, Abraham J, Ackerman I, Aggarwal R, Ahn SY, Ali MK, Alvarado M, Anderson HR, Anderson LM, Andrews KG, Atkinson C, Baddour LM, Bahalim AN, Barker-Collo S, Barrero LH, Bartels DH, Basáñez MG, Baxter A, Bell ML, Benjamin EJ, Bennett D (2012). Years lived with disability (YLDs) for 1160 sequelae of 289 diseases and injuries 1990–2010: a systematic analysis for the Global Burden of Disease Study 2010. Lancet.

[6] Hoy D, March L, Brooks P, Blyth F, Woolf A, Bain C, Williams G, Smith E, Vos T, Barendregt J, Murray C, Burstein R, Buchbinder R (2014). The global burden of low back pain: estimates from the Global Burden of Disease 2010 study. Ann Rheum Dis.

[7] Hoy D, March L, Woolf A, Blyth F, Brooks P, Smith E, Vos T, Barendregt J, Blore J, Murray C, Murray C, Burstein R, Buchbinder R (2014). The global burden of neck pain: estimates from the Global Burden of Disease 2010 study. Ann Rheum Dis.

[8] Raspe H (2012). Back pain - Health Monitoring of the Federal Government.

[9] Kleinman N, Patel AA, Benson C, Macario A, Kim M, Biondi DM (2014). Economic burden of back and neck pain: effect of a neuropathic component. Popul Health Manag.

[10] Martin BI, Deyo RA, Mirza SK, Turner JA, Comstock BA, Hollingworth W, Sullivan SD (2008). Expenditures and health status among adults with back and neck problems. JAMA.

[11] Schofield D, Kelly S, Shrestha R, Callander E, Passey M, Percival R (2012). The impact of back problems on retirement wealth. Pain.

[12] Wolff R, Clar C, Lerch C, Kleijnen J (2011). Epidemiology of chronic non-malignant pain in Germany. Schmerz.

[13] Apkarian AV, Baliki MN, Geha PY (2009). Towards a theory of chronic pain. Prog Neurobiol.

[14] Guez M, Hildingsson C, Nilsson M, Toolanen G (2002). The prevalence of neck pain: a population-based study from northern Sweden. Acta Orthop Scand.

[15] Bovim G, Schrader H, Sand T (1994). Neck pain in the general population. Spine (Phila Pa 1976).

[16] Balague F, Mannion AF, Pellise F, Cedraschi C (2012). Non-specific low back pain. Lancet.

[17] Scherer M, Plat E (2009). Nackenschmerzen: DEGAM Leitlinienset Nr. 13. 1. Auflage edn.

[18] Lewandowski W, Jacobson A (2013). Bridging the gap between mind and body: a biobehavioral model of the effects of guided imagery on pain, pain disability, and depression. Pain Manag Nurs.

[19] Bundesärztekammer (BÄK), Kassenärztliche Bundesvereinigung (KBV), Arbeitsgemeinschaft der Wissenschaftlichen Fachgesellschaften (AWMF) (2010). Nationale VersorgungsLeitlinie Kreuzschmerz – Langfassung.

[20] Savigny P, Kuntze S, Watson P, Underwood M, Ritchie G, Cotterell M, Hill D, Browne N, Buchanan E, Coffey P, Dixon P, Drummond C, Flanagan M, Greenough C, Griffiths M, Halliday-Bell J, Hettinga D, Vogel S, Walsh D (2009). Low Back Pain: Early Management of Persistent Non-Specific Low Back Pain.

[21] Dagenais S, Tricco AC, Haldeman S (2010). Synthesis of recommendations for the assessment and management of low back pain from recent clinical practice guidelines. Spine J.

[22] NIH (1996). Integration of behavioral and relaxation approaches into the treatment of chronic pain and insomnia. NIH technology assessment panel on integration of behavioral and relaxation approaches into the treatment of chronic pain and insomnia. JAMA.

[23] NIH (1995). Integration of behavioral and relaxation approaches into the treatment of chronic pain and insomnia. NIH technology assessment panel on integration of behavioral and relaxation approaches into the treatment of chronic pain and insomnia. NIH Technol Statement vol. Oct 16–18.

[24] Lee C, Crawford C, Hickey A, Active Self-Care Therapies for Pain Working G (2014). Mind-body therapies for the self-management of chronic pain symptoms. Pain Med.

[25] Sherman KJ, Cherkin DC, Wellman RD, Cook AJ, Hawkes RJ, Delaney K, Deyo RA (2011). A randomized trial comparing yoga, stretching, and a self-care book for chronic low back pain. Arch Intern Med.

[26] Sobel DS (2000). MSJAMA: mind matters, money matters: the cost-effectiveness of mind/body medicine. JAMA.

[27] Sobel DS (2000). The cost-effectiveness of mind-body medicine interventions. Prog Brain Res.

[28] Diezemann A (2011). Relaxation techniques for chronic pain. Schmerz.

[29] Astin JA (2004). Mind-body therapies for the management of pain. Clin J Pain.

[30] Benson H, Beary JF, Carol MP (1974). The relaxation response. Psychiatry.

[31] Bhasin MK, Dusek JA, Chang BH, Joseph MG, Denninger JW, Fricchione GL, Benson H, Libermann TA (2013). Relaxation response induces temporal transcriptome changes in energy metabolism, insulin secretion and inflammatory pathways. PLoS One.

[32] Dusek JA, Benson H (2009). Mind-body medicine: a model of the comparative clinical impact of the acute stress and relaxation responses. Minn Med.

[33] Lauche R, Materdey S, Cramer H, Haller H, Stange R, Dobos G, Rampp T (2013). Effectiveness of home-based cupping massage compared to progressive muscle relaxation in patients with chronic neck pain–a randomized controlled trial. PLoS One.

[34] Rosenzweig S, Greeson JM, Reibel DK, Green JS, Jasser SA, Beasley D (2010). Mindfulness-based stress reduction for chronic pain conditions: variation in treatment outcomes and role of home meditation practice. J Psychosom Res.

[35] van Tulder MW, Ostelo R, Vlaeyen JW, Linton SJ, Morley SJ, Assendelft WJ (2000). Behavioral treatment for chronic low back pain: a systematic review within the framework of the Cochrane Back Review Group. Spine.

[36] Posadzki P, Ernst E (2011). Guided imagery for musculoskeletal pain: a systematic review. Clin J Pain.

[37] Cramer H, Haller H, Lauche R, Dobos G (2012). Mindfulness-based stress reduction for low back pain. A systematic review. BMC Complement Altern Med.

[38] Kim DK, Rhee JH, Kang SW (2014). Reorganization of the brain and heart rhythm during autogenic meditation. Front Integr Neurosci.

[39] Statista: *Anzahl der Smartphone-Nutzer in Deutschland in den Jahren 2009 bis 2014 (in Millionen)*. [http://de.statista.com/statistik/daten/studie/198959/umfrage/anzahl-der-smartphonenutzer-in-deutschland-seit-2010/] (Accessed 12 August 2014)

[40] Statistisches Bundesamt (2013). Zahlen und Fakten: Bevölkerung.

[41] Rosser BA, Eccleston C (2011). Smartphone applications for pain management. J Telemed Telecare.

[42] Wallace LS, Dhingra LK (2014). A systematic review of smartphone applications for chronic pain available for download in the United States. J Opioid Manag.

[43] Witt CM, Chesney M, Gliklich R, Green L, Lewith G, Luce B, McCaffrey A, Rafferty Withers S, Sox HC, Tunis S, Berman BM (2012). Building a strategic framework for comparative effectiveness research in complementary and integrative medicine. Evid Based Complement Alternat Med.

[44] Kanji N, White AR, Ernst E (2006). Autogenic training for tension type headaches: a systematic review of controlled trials. Complement Ther Med.

[45] Kabat-Zinn J (1994). Wherever You Go, There You Are: Mindfulness Meditation in Everyday Life.

[46] Kabat-Zinn J (1982). An outpatient program in behavioral medicine for chronic pain patients based on the practice of mindfulness meditation: theoretical considerations and preliminary results. Gen Hosp Psychiatry.

[47] Kabat-Zinn J, Lipworth L, Burney R (1985). The clinical use of mindfulness meditation for the self-regulation of chronic pain. J Behav Med.

[48] Hassed C (2013). Mind-body therapies–use in chronic pain management. Aust Fam Physician.

[49] *Relaxation techniques for health: an introduction*. [http://nccam.nih.gov/health/stress/relaxation.htm]

[50] Huskisson EC, Scott J, Westhoff G (1993). VAS Visuelle Analog-Skalen; auch VAPS Visual Analogue Pain Scales, NRS Numerische Rating-Skalen; Mod. Kategorialskalen. Handbuch psychosozialer Meßinstrumente - ein Kompendium für epidemiologische und klinische Forschung zu chronischer Krankheit.

[51] Nilges P, Koster B, Schmidt CO (2007). Pain acceptance - concept and validation of a German version of the chronic pain acceptance questionnaire. Schmerz.

[52] Last J, Spasoff R, Harris S (2001). A Dictionary of Epidemiology.

[53] Witt CM, Manheimer E, Hammerschlag R, Ludtke R, Lao L, Tunis SR, Berman BM (2012). How well do randomized trials inform decision making: systematic review using comparative effectiveness research measures on acupuncture for back pain. PLoS One.

[54] Glasgow RE, Gaglio B, Bennett G, Jerome GJ, Yeh HC, Sarwer DB, Appel L, Colditz G, Wadden TA, Wells B (2012). Applying the PRECIS criteria to describe three effectiveness trials of weight loss in obese patients with comorbid conditions. Health Serv Res.

[55] Zwarenstein M, Treweek S, Gagnier JJ, Altman DG, Tunis S, Haynes B, Oxman AD, Moher D (2008). Improving the reporting of pragmatic trials: an extension of the CONSORT statement. BMJ.

[56] Thorpe KE, Zwarenstein M, Oxman AD, Treweek S, Furberg CD, Altman DG, Tunis S, Bergel E, Harvey I, Magid DJ, Chalkidou K (2009). A pragmatic-explanatory continuum indicator summary (PRECIS): a tool to help trial designers. CMAJ.

[57] Blödt S, Schützler L, Huang W, Pach D, Brinkhaus B, Hummelsberger J, Kirschbaum B, Kuhlmann K, Lao L, Liang F, Mietzner A, Mittring N, Müller S, Paul A, Pimapao-Niederle C, Roll S, Wu H, Zhu J, Witt CM (2013). Effectiveness of additional self-care acupressure for women with menstrual pain compared to usual care alone: using stakeholder engagement to design a pragmatic randomized trial and study protocol. Trials.

[58] Hoy DG, Protani M, De R, Buchbinder R (2010). The epidemiology of neck pain. Best Pract Res Clin Rheumatol.

[59] Cecchi F, Pasquini G, Paperini A, Boni R, Castagnoli C, Pistritto S, Macchi C (2014). Predictors of response to exercise therapy for chronic low back pain: result of a prospective study with one year follow-up. Eur J Phys Rehabil Med.

[60] Pillastrini P, Gardenghi I, Bonetti F, Capra F, Guccione A, Mugnai R, Violante FS (2012). An updated overview of clinical guidelines for chronic low back pain management in primary care. Joint Bone Spine.

[61] Bolton JE (1999). Accuracy of recall of usual pain intensity in back pain patients. Pain.

[62] Bolton JE, Humphreys BK, van Hedel HJ (2010). Validity of weekly recall ratings of average pain intensity in neck pain patients. J Manipulative Physiol Ther.

[63] Jensen MP, Wang W, Potts SL, Gould EM (2012). Reliability and validity of individual and composite recall pain measures in patients with cancer. Pain Med.

[64] Stone AA, Broderick JE, Shiffman SS, Schwartz JE (2004). Understanding recall of weekly pain from a momentary assessment perspective: absolute agreement, between- and within-person consistency, and judged change in weekly pain. Pain.

